# Retinoic Acid-Treated Pluripotent Stem Cells Undergoing Neurogenesis Present Increased Aneuploidy and Micronuclei Formation

**DOI:** 10.1371/journal.pone.0020667

**Published:** 2011-06-06

**Authors:** Rafaela C. Sartore, Priscila B. Campos, Cleber A. Trujillo, Bia L. Ramalho, Priscilla D. Negraes, Bruna S. Paulsen, Tamara Meletti, Elaine S. Costa, Leonardo Chicaybam, Martin H. Bonamino, Henning Ulrich, Stevens K. Rehen

**Affiliations:** 1 Instituto de Ciências Biomédicas, Universidade Federal do Rio de Janeiro, Rio de Janeiro, Rio de Janeiro, Brazil; 2 Departamento de Bioquímica do Instituto de Química, Universidade de São Paulo, São Paulo, São Paulo, Brazil; 3 Instituto de Puericultura e Pediatria Martagão Gesteira, Universidade Federal do Rio de Janeiro, Rio de Janeiro, Rio de Janeiro, Brazil; 4 Divisão de Medicina Experimental, Instituto Nacional de Câncer, Rio de Janeiro, Rio de Janeiro, Brazil; Federal University of Rio de Janeiro, Brazil

## Abstract

The existence of loss and gain of chromosomes, known as aneuploidy, has been previously described within the central nervous system. During development, at least one-third of neural progenitor cells (NPCs) are aneuploid. Notably, aneuploid NPCs may survive and functionally integrate into the mature neural circuitry. Given the unanswered significance of this phenomenon, we tested the hypothesis that neural differentiation induced by *all-trans* retinoic acid (RA) in pluripotent stem cells is accompanied by increased levels of aneuploidy, as previously described for cortical NPCs *in vivo*. In this work we used embryonal carcinoma (EC) cells, embryonic stem (ES) cells and induced pluripotent stem (iPS) cells undergoing differentiation into NPCs. Ploidy analysis revealed a 2-fold increase in the rate of aneuploidy, with the prevalence of chromosome loss in RA primed stem cells when compared to naïve cells. In an attempt to understand the basis of neurogenic aneuploidy, micronuclei formation and survivin expression was assessed in pluripotent stem cells exposed to RA. RA increased micronuclei occurrence by almost 2-fold while decreased survivin expression by 50%, indicating possible mechanisms by which stem cells lose their chromosomes during neural differentiation. DNA fragmentation analysis demonstrated no increase in apoptosis on embryoid bodies treated with RA, indicating that cell death is not the mandatory fate of aneuploid NPCs derived from pluripotent cells. In order to exclude that the increase in aneuploidy was a spurious consequence of RA treatment, not related to neurogenesis, mouse embryonic fibroblasts were treated with RA under the same conditions and no alterations in chromosome gain or loss were observed. These findings indicate a correlation amongst neural differentiation, aneuploidy, micronuclei formation and survivin downregulation in pluripotent stem cells exposed to RA, providing evidence that somatically generated chromosomal variation accompanies neurogenesis *in vitro*.

## Introduction

Pluripotent stem cells, as capable of forming any cell type in the body, have been extensively used to study developmental biology, including neurogenesis. In the beginning, embryonal carcinoma (EC) cells were the unique choice for analyzing differentiation *in vitro* [1]. With the isolation of embryonic stem (ES) cells [2] and more recently, induced pluripotent stem (iPS) cells derived from reprogrammed somatic cells [3], [4], [5], new tools for studying the stemness behavior and the ability to differentiate into neurons became available.

The *all-trans* retinoic acid (RA) is a metabolic compound derived from vitamin A, widely associated with neurogenesis [6]. During embryogenesis, RA contributes to patterning of the neural plate and neural tube [7], [8], [9]. In adulthood, it plays a role in neuronal differentiation within the dentate gyrus [10], in the maintenance of motor neurons [11] and in mammalian nerve regeneration [12]. Indeed, RA is the most used morphogen to produce neural progenitor cells (NPCs) and neurons from stem cells *in vitro* [13], [14], [15], [16].

The existence of variations in chromosome number (aneuploidy) in NPCs and neurons from mice, fish and humans has been previously described [17], [18], [19]. Aneuploid NPCs are present at a substantial rate of 33% in the developing brain [18], [20], [21] and 20% in the cerebellum [22]. Also, aneuploid neurons can be found functionally integrated into the neural circuitry of adults [21], [23]. Even though the significance of aneuploidy within the nervous system is still unknown, the brain is a mosaic composed by euploid and aneuploid cells, which may contribute for brain complexity [18].

Despite chromosomal mosaicism has been extensively described within the brain *in vivo*, possible causes of aneuploidy during development of the central nervous system (CNS) have never been explored. Here, we asked whether neurogenesis triggered by RA *in vitro* is accompanied by aneuploidy in pluripotent stem cell types.

## Materials and Methods

### Reprogramming into pluripotent stem cells

Induced pluripotent stem (iPS) cells were developed in house [24] as previously described by Takahashi and Yamanaka [3]. The procedures were according to regulations set by international guidelines [25] and the local Biosafety Committee. Mouse embryonic fibroblasts (MEF) isolated from C57Black6 mice were transduced with retroviral vectors containing the human cDNAs of Oct4, Sox2, Klf4 and c-Myc inserted into moloney-based retroviral vectors (pMXs). 293T cells [26] (kindly provided by Dr. Bryan Strauss from Instituto do Coração, INCOR, Universidade de São Paulo) were used as packaging cells to the virus production after calcium phosphate based transfection with the following plasmids: pMXs (20 µg), pMDM (encoding mlv GAG/POL, 10 µg) and pMD.G (encoding VSV G, 6 µg). The viral supernatants were collected 48 and 72 h post-transfection, filtered (0.22 µm) and concentrated at 32,000 g for 60 min. MEF were exposed to two rounds of transduction with the 293T concentrated supernatants. To improve transduction, polybrene (Sigma-Aldrich Corp., St. Louis, USA) was used at 8 µg/ml and the plate was centrifuged at 540 g for 45 min. Two days post-transduction, the cells were cultured in iPS cells-medium (detailed below) and treated with valproic acid (1 mM, Sigma-Aldrich Corp., St. Louis, USA) for one week. Three days after transduction, 2.5×10^5^ cells were plated on 2% gelatin-coated 35 mm plate with mitotically inactivated MEF as feeders. Mouse ES cells-like colonies were selected 15 days after the transduction. Valproic acid stock solutions were made in phosphate buffered saline (PBS).

### Cell culture

#### EC cells

P19 embryonal carcinoma cells [27] generously donated by Dr. Michael McBurney from Ottawa Hospital Research Institute (University of Ottawa) were cultured in Dulbecco's Modified Eagle's Medium (DMEM) (Invitrogen, Life Technologies, Carlsbad, USA) supplemented with 10% fetal bovine serum (FBS) (Cultilab, Campinas, Brazil), 100 units/ml penicillin, 100 µg/ml streptomycin, 2 mM L-glutamine and 2 mM sodium pyruvate (all from Sigma-Aldrich Corp., St. Louis, USA), in a humidified incubator at 5% CO_2_ and 37°C.

#### ES and iPS cells

E14TG2a mouse embryonic stem (ES) cells [28], [29] (kindly provided by Dr. Joshua Brickman from the Institute for Stem Cell Research, MRC Centre for Regenerative Medicine, University of Edinburgh) were grown on gelatin-coated tissue culture dishes in Glasgow Minimum Essential Medium (GMEM) (Cultilab, Campinas, Brazil) supplemented with 15% FBS (Cultilab, Campinas, Brazil), 2 mM L-glutamine, 55 mM 2-mercaptoethanol and 1% non-essential amino acids (all from Invitrogen, Life Technologies, Carlsbad, USA). Mouse iPS cells were cultured in high-glucose DMEM/F12 supplemented with 15% Knockout™ Serum Replacement (KSR), 2 mM L-glutamine, 55 mM 2-mercaptoethanol and 1% non-essential amino acids (all from Invitrogen, Life Technologies, Carlsbad, USA). Mitotically inactivated MEF were used as feeders for iPS cells cultures. Conditioned medium (1∶500) (v∶v) from chinese hamster ovary (CHO) cells carrying leukemia inhibitory factor (LIF)-encoding vector was used as source of LIF for ES and iPS cells maintenance. Cells were cultured in a humidified incubator at 5% CO_2_ and 37°C.

Since the use of enzymes (to obtain single-cell suspensions) may affect the karyotype status of embryonic stem cells [30], [31], we have adopted manual dissection (which produces cell clumps, instead of single-cells) to handle our cells and routinely monitored them for karyotypic changes. While ES cells exhibited the same number of chromosomes (40) after dozens of passages, iPS cells presented a mixed population formed by cells with 40 or 41 chromosomes even after 3 passages, as similarly described for human iPS cells [32].

### Neural differentiation

#### EC cells

EC cells were differentiated into NPCs as previously described [15], [33], [34]. Briefly, 5×10^5^ cells/ml were transferred to non-adherent dishes to form embryoid bodies (EBs). EBs developed in defined medium (DMEM supplemented with 2 mM L-glutamine, 2 mM sodium pyruvate, 2.4 mg/ml sodium bicarbonate, 5 µg/ml insulin, 30 µg/ml human apo-transferrin, 20 µM ethanolamine, 30 nM sodium selenite, 100 U/ml penicillin, 100 µg/ml streptomycin and 10 mM Hepes, pH 7.2) supplemented with 1 µM *all-trans* retinoic acid (RA) (Sigma-Aldrich Corp., St. Louis, USA) dissolved in dimethyl sulfoxide (DMSO) (Sigma-Aldrich Corp., St. Louis, USA). After 2 days in the presence of RA, EBs were collected and plated onto adherent culture flasks with DMEM supplemented with 10% FBS for 48 h. DMSO (0.8%) was used as vehicle.

#### ES and iPS cells

ES and iPS cells differentiation into NPCs was carried out by RA treatment as previously described [35], [36]. EBs were formed after transferring 2.5×10^5^ cells/ml to non-adherent dishes. EBs were cultured in medium with FBS without LIF. The percentage of FBS was 20% for ES-EBs and 15% for iPS-EBs. After 4 days of formation, EBs were exposed to 5 µM RA for additional 4 days. RA stock solution was prepared in DMSO, which was also added to the control groups at the final concentration of 0.01%. For immunofluorescence assays, EBs after 8 days *in vitro* were dissociated in 0.05% trypsin/EDTA and cells were plated onto precoated polyornithine (100 µg/ml, Sigma-Aldrich Corp., St. Louis, USA) and laminin (5 µg/ml, Invitrogen, Life Technologies, Carlsbad, USA) coverslips. After 2 h of plating, immunofluorescence was performed as below.

For analysis of DNA content and neural immunophenotyping, EBs exposed to RA or vehicle were dissociated as mentioned above. Then, cells were plated at a density of 12×10^5^ cells/cm^2^ onto polyornithine and laminin-coated plates and maintained in neuronal medium, composed by high-glucose DMEM/F12 supplemented with 2% N2, 20 ng/ml fibroblast growth factor-2 (FGF-2) and 55 mM 2-mercaptoethanol (all from Invitrogen, Life Technologies, Carlsbad, USA) for 5 days, then DNA analysis by flow cytometry was carried out. For further analyses, EBs were also plated into polyornithine and laminin substrates and maintained in neuronal medium for 5 days.

### Immunofluorescence

Cells were fixed with 4% paraformaldehyde in PBS for 20 min. Blocking was achieved after 20 min of incubation with 3% bovine serum albumin (BSA) combined with 0.1% Triton X-100 (Sigma-Aldrich Corp., St. Louis, USA) in PBS. Cells were then incubated for 2 h with primary antibodies diluted in PBS plus 0.1% Triton X-100 and 3% BSA. Primary antibodies for nestin (Chemicon, Millipore, Temecula, USA) and βIII-tubulin (Santa Cruz Biotechnology, Santa Cruz, USA) were used. After rinsing with PBS, anti-mouse Alexa 546-conjugated and anti-rabbit Alexa 488-conjugated secondary antibodies (Life Technologies, Carlsbad, USA) were added for 1 h, followed by 5 min incubation with DAPI (4′-6-diamidino-2-phenylindole). After rinsing with PBS, coverslips were mounted on slides with Vectashield (Vector Laboratories, Burlingame, USA) and examined on an Axiovert-200 epifluorescence microscope (Carl Zeiss, Göttingen, Germany).

### Chromosome counts

Metaphase spreads were prepared as previously described [37]. Cells were incubated with 0.1 µg/ml KaryoMAX colcemid (Gibco Invitrogen, Life Technologies, Carlsbad, USA) for 6 h, then dissociated using trypsin/EDTA 0.05% and incubated for 15 min in a 75 mM KCl hypotonic solution at 37°C. The swollen cells were fixed in a methanol/glacial acetic acid 3∶1 (v∶v) solution (Merck KGaA, Darmstadt, Germany) and stored at 4°C overnight. On the next day, cell suspension was washed with the fixative solution and then spread onto glass slides. The slides were quickly exposed to water steam to burst the cells. Chromosomes were stained with DAPI, and the slides coverslipped with n-propyl galate. The mounted slides were examined on a fluorescence microscope (Nikon Eclipse 80i, Nikon Instruments Inc., NY, USA). At least 60 metaphases were counted on each condition per assay.

Once iPS cells present a mixed population formed by cells with 40 or 41 chromosomes, we adopted this interval as the modal chromosome number for this cell line. Chromosome numbers below and above range were considered as an increase in the aneuploidy rate for iPS cells.

### TUNEL assay

Apoptosis was determined in NPCs derived from EC cells using an apoptosis detection kit (*In Situ* Cell Death Detection Kit, Fluorescein; Roche Applied Science, Indianapolis, USA). After harvesting, cells were fixed in 2% paraformaldehyde in PBS for 30 min at 4°C, rinsed with PBS and resuspended in 0.2% Triton-X-100 and 0.2% Tween in PBS for 10 min at 4°C. Then, cells were rinsed again with PBS and incubated with TUNEL reaction mixture as suggested by the manufacturer. For negative controls, instead of the TUNEL reaction mixture, cells were incubated with label solution (without terminal transferase). For positive controls, cells were incubated with DNase I (3 U/µl, Ambion, Austin, USA) for 10 min at room temperature. Cells were analyzed by flow cytometry (Beckman Coulter, Fc500) and data were analyzed using the WinMDI 2.8 software available at http://facs.scripps.edu/software.html. Thirty thousand events were acquired per sample with fluorescence measured in logarithmic scales.

For apoptosis evaluation in NPCs derived from ES cells, ApopTag Plus Fluorescein *In Situ* Apoptosis Detection Kit (Chemicon, Temecula, USA) was used according to the manufacturer's instructions. Briefly, EBs were dissociated and filtered through a 40 µm nylon cell strainer (BD Biosciences, San Jose, USA), cells were fixed in 1% paraformaldehyde in PBS for 15 min at 4°C, rinsed in PBS and resuspended in 70% ice-cold ethanol for at least 2 h. Then, cells were incubated with TdT enzyme and anti-digoxigenin fluorescein conjugate. As negative control, cells were not incubated with enzyme but with anti-digoxigenin fluorescein. Cells were analyzed by flow cytometry (BD FACSCanto II, BD Biosciences, San Jose, USA) and data were analyzed using the Summit software (Dako, Glostrup, Denmark). Twenty thousand events were acquired per sample with fluorescence measured in logarithmic scales.

### Flow cytometric analysis of DNA content

The DNA content of NPCs derived from EC cells was determined by flow cytometry. After harvesting, cells were fixed in 70% ethanol at −20°C for at least 2 h. Then, cells were rinsed twice with PBS, stained with propidium iodide (50 µg/ml, Sigma-Aldrich Corp., St. Louis, USA) in the presence of RNase (100 µg/ml, Ambion, Austin, USA) for 1 h at 37°C and taken to the cytometer (Beckman Coulter, Fc500, Fullerton) for DNA content analysis as previous described by Capparelli *et al.* [38]. Chick erythrocyte nuclei (CEN) were used at one-tenth of sample concentration as internal control. Thirty thousand events were acquired per sample with fluorescence measured in logarithmic scales. For determining changes in DNA content, relative DNA content was expressed as the ratio of G0/G1 peak to the mean of the CEN peak.

For analysis of DNA content within neuronal cells, RA treated-EBs derived from ES cells, dissociated and plated for 5 days, were fixed in ice-cold ethanol, rinsed with PBS and permeabilized with 0.5% Tween (Sigma-Aldrich Corp., St. Louis, USA) for 5 min. To prevent nonspecific immunolabeling, 5% BSA was used for 1 h. Then, samples were incubated on ice with primary antibody for βIII-tubulin (Sigma-Aldrich Corp., St. Louis, USA) for 1 h. After rinsing twice in PBS, anti-mouse Alexa 488-conjugated secondary antibody (Life Technologies, Carlsbad, USA) was added for 30 min on ice. Following PBS washing, propidium iodide (PI) mix consisting of 20 µg/ml PI (Sigma-Aldrich Corp., St. Louis, USA), 100 µg/ml RNase (Ambion, Austin, USA) and 0.1% Triton X-100 (Sigma-Aldrich Corp., St. Louis, USA) diluted in PBS was added and the cells were taken to the cytometer (BD FACSCanto II, BD Biosciences, San Jose, USA). Mouse lymphocytes stained under the same conditions were collected to calibrate the cytometer regarding the position of G0/G1 peak. Fifty thousand events were acquired per sample with fluorescence measured in logarithmic scales. Relative DNA content between positive and negative cells was expressed as the ratio of PI mean fluorescence intensity (MFI) of G0/G1 peak of positive cells in RA group to MFI of G0/G1 peak of negative cells in vehicle group.

### Micronuclei detection

For micronuclei detection, colonies of ES and iPS cells were exposed to 5 µM RA for 4 days. Cytochalasin B (6 µg/ml, Sigma-Aldrich Corp., St. Louis, USA) was added in the last 24 h of RA treatment. Cells were dissociated and fixed in methanol/glacial acetic acid 3∶1 (v∶v) (Merck KGaA, Darmstadt, Germany) solution, then placed onto slides and stained with 10% Giemsa (Merck KGaA, Darmstadt, Germany) diluted in distilled water (v∶v) for 30 min. At least 2,000 nuclei were counted per sample at 100× magnification. Differences in micronuclei fold change were relative to cells treated with vehicle.

### Quantitative RT-PCR

Total RNA was extracted from EBs after treatment with RA by using Trizol (Invitrogen, Life Technologies, Carlsbad, USA). DNAse I treatment (Apllied Biosystems, Life Technologies, Foster City, USA) was applied and 1 µg of total RNA was reverse-transcribed using cDNA Reverse Transcription Kit (Apllied Biosystems, Life Technologies, Foster City, USA) according to the manufacturer's instructions. Quantitative RT-PCR was performed using Power SYBR® Green PCR Master Mix (Apllied Biosystems, Life Technologies, Foster City, USA) on the 7500 Real-Time PCR System detector (Apllied Biosystems, Life Technologies, Foster City, USA). RT-PCR was performed for 45 cycles with primers derived from survivin (74 bp, 5′-GGCTGGGAACCCGATGAC-3′ and 5′- CGGTTAGTTCTTCCATCTGCTTCT -3′) and β-actin (85 bp, 5′-CATCACTATTGGCAACGAGCG-3′ and 5′-ATGGATGCCACAGGATTCCA-3′). All quantitative RT-PCR assays were carried out in triplicates from three independent experiments. Expression levels of individual transcripts were normalized to β-actin expression and fold differences were calculated using the 2^−ΔΔCt^ method.

### Protein Extraction, SDS-PAGE and Western Blotting

Cell lysates were obtained with lysis buffer (1% NP-40, 0.25% sodium deoxycholate, 50 mM Tris-HCl pH 7.4, 150 mM NaCl, 2 mM EDTA, 1 mM Na_3_VO_4_) supplemented with 1× phosphatase and protease inhibitors (Sigma-Aldrich Corp., St. Louis, USA). Cells were maintained in lysis buffer on ice and homogenized by shaking at 5 min intervals until its complete dissociation. Then, samples were centrifuged, the supernatants recovered and protein quantity was measured by Bradford method with albumin as standard. Forty µg of protein in sample buffer were boiled for 3 min and separated by SDS-PAGE in a 15% polyacrylamide gel at a constant voltage of 100 V. After separation, proteins were transferred into a nitrocellulose membrane (Hybond, Amersham, Piscataway, USA) in a semi-dry system (Bio-Rad, Hercules, USA) for 120 min at constant amperage of 0.1 mA.

For nonspecific blocking, 5% nonfat milk in tris-buffered saline and 0.05% Tween-20 (TBS-T) was added for 2 h under agitation at room temperature. After incubation with primary antibodies for survivin (Sigma-Aldrich Corp., St. Louis, USA) or α-tubulin (Santa Cruz Biotechnology, Santa Cruz, USA) overnight at 4°C, membranes were washed and probed with anti-rabbit or anti-mouse peroxidase-conjugated secondary antibodies (Invitrogen, Life Technologies, Carlsbad, USA) for 2 h under agitation at room temperature. Antibodies were diluted in 5% nonfat milk in TBS-T. Then, membranes were washed in TBS-T. Blots were revealed with SuperSignal West Pico (Pierce, Rockford, USA). The resulting bands were measured with ImageJ software. The values were expressed in arbitrary units while the survivin level was plotted in relation to α-tubulin in the same lane. The normalization was made considering vehicle as 1.

### Statistical analysis

Results were expressed as mean ± S.E.M. Analysis of statistical significance was performed using 4.0 GraphPad Prism software. Data were analyzed by unpaired t-test.

## Results

### Chromosome instability is increased in NPCs derived from EC cells treated with RA

**Figure 1:**
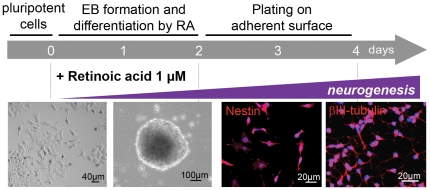
Neural differentiation in EC cells after RA treatment. EC, embryonal carcinoma cells; RA, *all-trans* retinoic acid.

In order to investigate whether neural differentiation is accompanied by aneuploidy in EC cells, EBs were formed and concomitantly exposed to RA to trigger their differentiation into NPCs (RA-NPCs). On day 4, immunofluorescence revealed nestin and βIII-tubulin in, respectively, 96.4±2.3% and 94.3±3.6% of RA primed cells, assuring their neural identity after the treatment (figure 1).

To identify changes in chromosome number, metaphases were obtained from RA-NPCs and untreated cells. As previously reported, EC cells possess massive genomic instability even in the undifferentiated state [39]. Since we found that 89.0±0.6% of undifferentiated EC cells presented between 59 and 65 chromosomes (data not shown), we considered this interval as the basal level of chromosomal instability in this lineage. Chromosome numbers below and above this range were considered as an increase in aneuploidy for EC cells.

**Figure 2:**
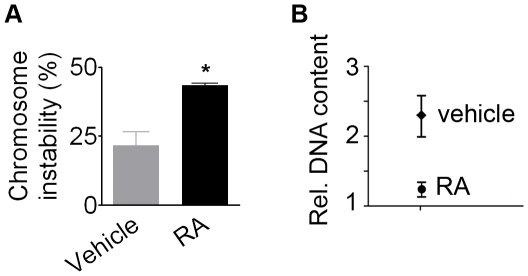
Chromosomal instability increases after induction of neural phenotype by RA in EC cells. (A) The rate of chromosomal instability presented a 2-fold increase. The bars indicate mean ± S.E.M. of two independent assays, *p<0.05. (B) Analysis of relative DNA content demonstrated that neural cells (RA) presented less DNA than cells incubated with vehicle. RA, *all-trans* retinoic acid.

EC-derived RA-NPCs displayed a 2-fold increase (44.5±0.6%) in numerical chromosomal instability when compared to EC cells incubated with vehicle (21.6±4.9%) (figure 2A). Moreover, analysis of DNA content by flow cytometry revealed that RA-NPCs displayed less DNA content than EC cells incubated with vehicle (figure 2B), indicating loss of chromosomes after RA treatment.

### Aneuploidy is increased in NPCs derived from ES and iPS cells after treatment with RA

In order to evaluate whether RA also increases aneuploidy in NPCs derived from chromosomal stable stem cells, NPCs were derived from both ES and iPS cells.

**Figure 3:**
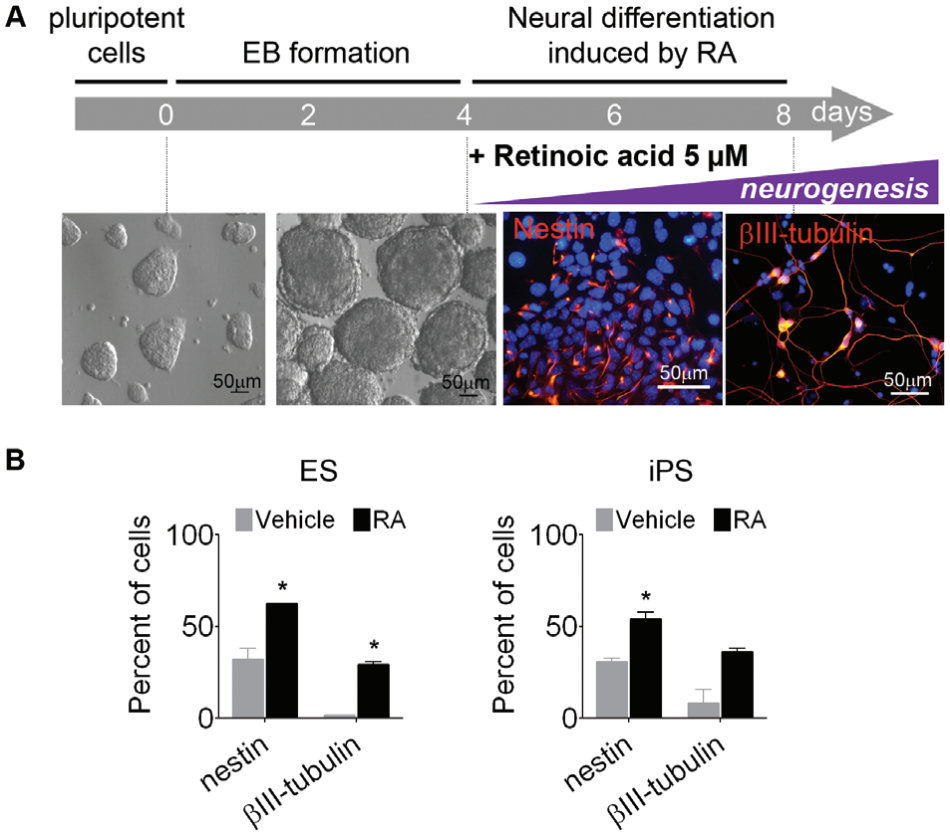
Neural differentiation in ES and iPS cells after RA treatment. (A) Schematic representation of protocol. (B) Immunofluorescence quantification confirmed commitment into neural progenitors (nestin) and young neurons (βIII-tubulin). The images correspond to ES-differentiated cells. The bars indicate mean ± S.E.M. of two independent assays, *p<0.05. ES, embryonic stem cells; iPS, induced pluripotent stem cells; RA, *all-trans* retinoic acid.

Neural differentiation of ES and iPS cells was carried out as previously described [35], [36]. On day 8, nestin and βIII-tubulin were present, respectively, in 62.7±0.1% and 29.0±1.6% in RA primed ES cells and in 54.0±3.9% and 36±2.1% in RA primed iPS cells (figure 3).

**Figure 4:**
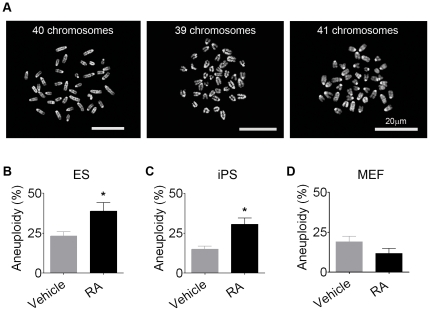
RA induces aneuploidy increase in pluripotent stem cells but not in somatic cells. (A) Metaphase spreads of RA-NPCs derived from ES cells stained with DAPI. After RA-neural differentiation, the aneuploidy level increased in (B) ES and (C) iPS cells, while (D) mouse embryonic fibroblasts (MEF) exposed to RA did not present aneuploidy increase. The data refer to mean ± S.E.M. of three independent experiments, *p<0.05. ES, embryonic stem cells; iPS, induced pluripotent stem cells; NPCs, neural progenitor cells; RA, *all-trans* retinoic acid.

Analysis of the chromosomal complement of RA-treated ES cells (figure 4A) demonstrated that aneuploidy increased from 23.3±2.7% in cells incubated with vehicle only to 38.7±5.4% in cells undergoing differentiation by RA (figure 4B). The same phenomenon was demonstrated for RA-treated iPS cells, which presented a 2-fold increase in the aneuploidy rate when compared to cells incubated with vehicle (31.5±3.8% compared to 14.0±1.4%) (figure 4C). On the other hand, the parental MEF with 40 chromosomes, from which our iPS cells were generated, did not show any increase in aneuploidy rate when exposed to RA under the same conditions as ES and iPS cells (5 µM RA for 4 days) (figure 4D).

**Figure 5:**
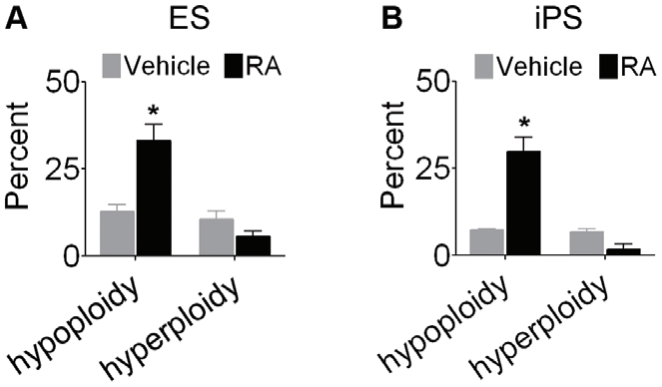
Hypoploidy is the main kind of aneuploidy in RA-NPCs. Upon (A) ES cells and (B) iPS cells differentiation with RA, the hypoploidy level increased, whereas hyperploidy level was not altered. The data refer to mean ± S.E.M. of three independent experiments, *p<0.05. ES, embryonic stem cells; iPS, induced pluripotent stem cells; RA-NPCs, neural progenitor cells derived from *all-trans* retinoic acid treatment.

Neural commitment with RA revealed that chromosome loss (hypoploidy) was more prevalent than chromosome gain (hyperploidy) in pluripotent stem cells. In ES-derived RA-NPCs, 33.1±4.6% of the cells were hypoploid compared to 12.7±2.0% in cells incubated with vehicle, whereas the proportion of hyperploidy was not increased by RA (10.5±2.4% in vehicle compared to 5.6±1.5% in RA) (figure 5A). In iPS-derived RA-NPCs, hypoploidy was again prevalent (29.8±4.2% in RA compared to 7.3±0.4% in vehicle) while hyperploidy was not increased by RA (6.7±1.0% in vehicle compared to 1.6±1.6% in RA) (figure 5B). Table 1 shows the most common aneuploidies by chromosome number in RA-treated and untreated ES and iPS cells.

**Table 1:** Most common aneuploidies observed in pluripotent stem cells after RA treatment.

	Frequency (%)			
	ES		iPS	
Number of chromosomes	Vehicle	RA	Vehicle	RA
27	0,0	1,1	0,0	0,5
28	0,0	1,8	0,0	0,0
31	0,0	0,0	0,0	1,5
32	0,5	1,1	0,0	1,1
33	1,2	3,3	0,0	4,5
35	0,5	1,8	0,5	1,1
36	0,6	1,8	0,0	1,5
38	0,5	3,0	0,5	6,3
39	7,4	13,5	4,0	10,7
40	76,7	61,3	5,4	14,6
41	7,8	4,5	80,6	54,0
42	0,6	0,7	5,9	1,6

ES, embryonic stem cells; iPS, induced pluripotent stem cells; RA, *all-trans* retinoic acid.

**Figure 6:**
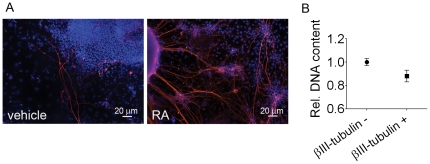
After RA treatment ES-derived neural cells exhibit loss of DNA content. (A) RA-treated EBs plated for 5 days are enriched in βIII-tubulin labeled young neurons forming a network with long processes. (B) Flow cytometry analysis of relative DNA content demonstrates decreased DNA content in cells expressing βIII-tubulin compared to cells negative for this marker. Data was expressed as the ratio of propidium iodide mean fluorescence intensity (MFI) of G0/G1 peak of positive cells in RA group to MFI of G0/G1 peak of unlabeled cells in vehicle group. The data refer to mean ± S.E.M. of four independent experiments, *p<0.1.

In order to verify whether the RA-increased aneuploidy was more prevalent in neural cells than in non-neural cells, we evaluated DNA content by flow cytometry in cells expressing or not βIII-tubulin. RA-treated EBs, dissociated and plated for 5 days, presented 47.0±6.4% of young neurons with long processes expressing βIII-tubulin (figure 6A). To identify decreases in DNA content, we assessed the PI mean fluorescence intensity (MFI). Young neurons expressing βIII-tubulin presented less DNA content than non-neural cells (figure 6B).

### Aneuploid NPCs are not prone to cell death

**Figure 7:**
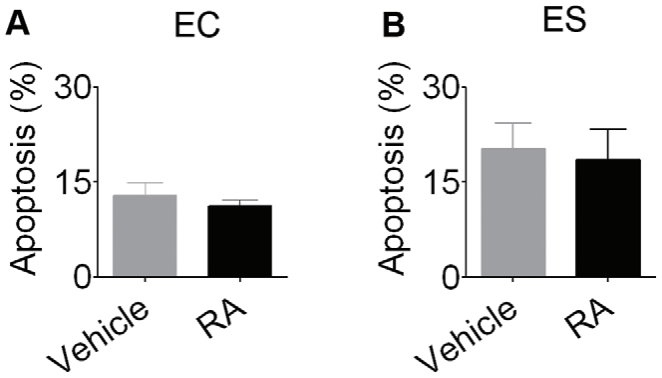
RA-treated stem cells do not necessarily undergo apoptosis. No difference in apoptosis between RA-NPCs and cells incubated with vehicle was observed in (A) EC cells or (B) ES cells. The bars indicate mean ± S.E.M. of three independent assays, *p<0.05. RA-NPCs, neural progenitor cells derived from *all-trans* retinoic acid treatment; EC, embryonal carcinoma cells; ES, embryonic stem cells.

Next, we asked whether aneuploid RA-NPCs would be prone to cell death. Both EC and ES cells undergoing neural differentiation by RA were analyzed for DNA fragmentation. TUNEL technique followed by flow cytometry demonstrated no difference in apoptosis levels between RA-NPCs and cells incubated with vehicle (figure 7A and B), demonstrating that an increase in aneuploidy is not necessarily followed by cell death in RA-treated stem cells.

### Micronuclei formation is increased in pluripotent stem cells after RA treatment

**Figure 8:**
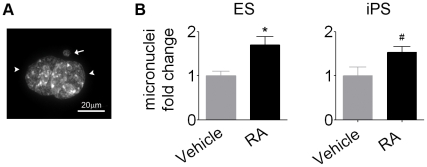
Micronuclei formation increase in pluripotent ES and iPS cells after RA exposure. (A) Photomicrography of two daughter cells nuclei (arrowhead) and a micronucleus (arrow) detected by DAPI staining. (B) RA increased micronuclei formation in ES and iPS cells. Data was expressed as the ratio of the mean of RA to the mean of vehicle. Bars indicate mean ± S.E.M. of three independent assays, *p<0.05, #p<0.1. ES, embryonic stem cells; iPS, induced pluripotent stem cells; RA, *all-trans* retinoic acid.

During mitosis, micronuclei can be formed when a chromosome does not appropriately attach to the mitotic spindle and become excluded from daughter cells nuclei. Micronucleus is an indicative of mitotic checkpoint failure and a possible mechanism by which aneuploidy emerges [40]. Our results revealed that RA-treated ES cells presented micronuclei scoring 1.7±0.2 times higher than cells incubated with the vehicle. In iPS cells, treatment with RA also resulted in micronuclei increase (1.5±0.1 times higher than vehicle) (figure 8).

### Survivin expression is reduced in RA-treated embryonic stem cells undergoing neurogenesis

**Figure 9:**
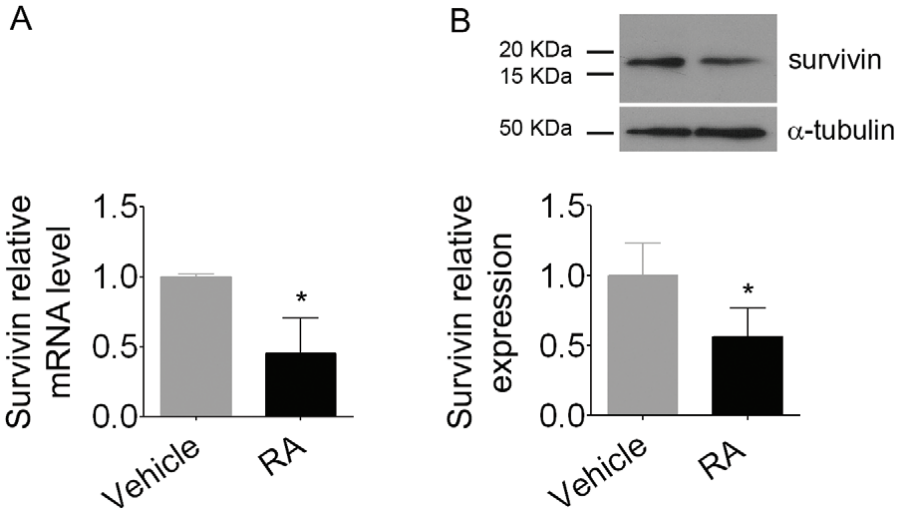
Reduced survivin expression in RA-treated pluripotent stem cells. (A) Survivin mRNA and (B) survivin protein were significantly reduced after RA treatment. For quantitative PCR, relative mRNA levels represent the amount of survivin mRNA compared to β-actin mRNA; for western blotting, relative survivin levels are compared to α-tubulin. Bars indicate mean ± S.E.M. of three independent assays, * p<0.1. Data were expressed as the ratio of the mean of RA to the mean of vehicle.

Since a downregulation of survivin correlates with RA exposure [41] and micronuclei formation in cancer cell lines [42], we decided to investigate whether survivin expression could also be reduced in embryonic stem cells undergoing neurogenesis after treatment with RA. Quantitative real-time PCR (figure 9A) and western blotting (figure 9B) analysis revealed that RA-treated cells presented a 50% reduction in survivin mRNA and protein levels when compared to untreated cells. These results indicate that a member of the chromosomal passenger complex may be involved in the mechanism by which RA increases aneuploidy and micronuclei formation in pluripotent stem cells.

## Discussion

It has been suggested that aneuploidy in the developing cerebral cortex is the consequence of chromosome missegregation and multipolar spindles [18], [43]. Hypoploidy is the prevalent form of aneuploidy observed during neurogenesis *in vivo* [18], [19], [20]. Here we show that an increase in aneuploidy, especially chromosome loss, is also observed in pluripotent stem cells undergoing neurogenesis *in vitro*.

Recently, aneuploidy was described in neural progenitor cells derived from the human fetal brain (hNPCs) after long-term cell culture [44]. Sareen and colleagues reported that long-term cultured hNPCs are susceptible to chromosome gains. They observed that after 9 weeks and 25 weeks *in vitro*, five NPCs lines and one NPC line, out of 21 cell lines, acquired trisomy 7 and trisomy 19, respectively.

While Sareen *et al.* described chromosome gain upon selective pressure *in vitro*, which emphasizes the need for careful cytogenetic evaluation of NPCs before their use in clinical or basic science, our data point to an increase in chromosome loss during neurogenesis *in vitro*, similarly to the observed *in vivo* [18]. Moreover, chromosome loss observed in pluripotent stem cells after retinoic acid treatment occurs independently of the number of passages, differently from Sareen *et al.*, in which NPCs must be cultured for at least 9 weeks to spontaneously generate chromosome gains.

A crucial question is whether aneuploidy here described was an authentic characteristic of neural commitment of pluripotent stem cells or a corollary effect of RA treatment. In this sense, MEF exposed to the same concentration of RA for the same period of time did not present numerical chromosome alterations. These results suggest at least 2 interpretations: 1) aneuploidy promoted by RA is associated to neural differentiation; 2) somatic cells are not prone to aneuploidy triggered by RA. In fact, embryonic stem cells exhibit rapid cycles of cell division by bypassing certain checkpoints [45], [46], [47], which is postulated to be a property of stemness [48] but could also make these cells more prone to aneuploidy.

It was previously reported that RA induces apoptosis in EC and ES cells [49], [50]. However, in both studies RA triggered apoptosis immediately upon its exposure, which was attenuated after cells start to differentiate [51]. In our case, apoptosis was measured in embryoid bodies at least 2 days after RA treatment, when cells were already committed to the neural fate. No difference in cell death was detected, indicating that an increase in aneuploidy is not directly associated with elimination of pluripotent stem cells undergoing neural differentiation *in vitro*.

Another issue addressed in this work was the description of a possible mechanism by which aneuploidy is triggered in pluripotent stem cells after RA treatment. Micronuclei reflect an inability to repair DNA lesions or deficits in checkpoints [40], [52]. ES and iPS cells exposed to RA presented an increase in micronuclei formation when compared to naïve cells. Consistently with our findings, Yang and coworkers reported micronucleation in NPCs *in vivo* [43]. It is worth mentioning that NPCs derived from the developing cerebral cortex show tolerance to chromosomal instability associated with an inefficient checkpoint mechanism [53], similarly to the observed in pluripotent stem cells [48]. Interestingly, the expression of survivin, which exerts multiple roles in chromosome segregation [54], [55], was reduced in embryonic stem cells after RA treatment.

It is well known that the depletion of survivin, functioning as tension-sensor at kinetochores-microtubules interactions in mitotic checkpoint [54], leads to unaligned chromosomes, failure in the mitotic checkpoint arrest [56] and inaccurate chromosome segregation [57], [58]. Indeed, in breast cancer cell lines, Pratt *et al.* demonstrated reduced expression of survivin in response to RA [41], while delayed expression of survivin results in cancer cells with micronuclei [42].

**Figure 10:**
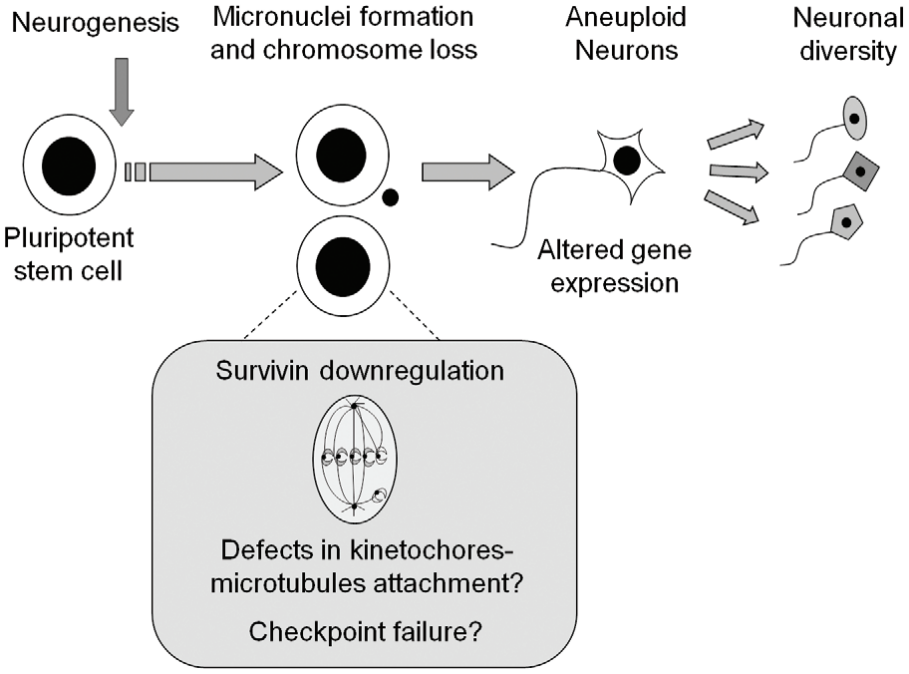
During neurogenesis, stem cells are prone to chromosome loss and micronuclei formation, which correlates with survivin downregulation. Neural aneuploidy may contribute to cell diversity within the CNS. CNS, central nervous system.

These data provide evidence that somatically generated chromosomal variation accompanies neurogenesis. We suggest that pluripotent stem cells undergoing neural differentiation are prone to mitotic checkpoint failure and defects in kinetochores-microtubules attachment, as indicated by the reduction in survivin expression, culminating with micronuclei formation and aneuploidy (figure 10). We cannot assure if aneuploidy is a cause or a consequence of neural differentiation but our results indicate that loss of entire chromosomes is a common feature of neurogenesis induced *in vitro*, which may lead to altered gene expression [20].

These results support the idea that aneuploidy is a common feature of neural progenitor cells both *in vivo* and *in vitro* and may play a crucial role in neurogenesis. Nevertheless, alternative protocols, without using RA, to induce neural differentiation should be applied to characterize a broader association between aneuploidy and neurogenesis. To our knowledge this is the first demonstration that aneuploidy and micronuclei are amplified during neurogenesis induced by RA in pluripotent stem cells. The physiological consequences of neurogenic aneuploidy should be carefully considered before exploring the full potential of neural cells derived *in vitro* towards therapy for neurodegenerative diseases.

## References

[1] Martin GR, Evans MJ (1974). The morphology and growth of a pluripotent teratocarcinoma cell line and its derivatives in tissue culture.. Cell.

[2] Evans MJ, Kaufman MH (1981). Establishment in culture of pluripotential cells from mouse embryos.. Nature.

[3] Takahashi K, Yamanaka S (2006). Induction of pluripotent stem cells from mouse embryonic and adult fibroblast cultures by defined factors.. Cell.

[4] Maherali N, Sridharan R, Xie W, Utikal J, Eminli S (2007). Directly reprogrammed fibroblasts show global epigenetic remodeling and widespread tissue contribution.. Cell Stem Cell.

[5] Mikkelsen TS, Hanna J, Zhang X, Ku M, Wernig M (2008). Dissecting direct reprogramming through integrative genomic analysis.. Nature.

[6] Maden M (2007). Retinoic acid in the development, regeneration and maintenance of the nervous system.. Nat Rev Neurosci.

[7] Diez del Corral R, Storey KG (2004). Opposing FGF and retinoid pathways: a signalling switch that controls differentiation and patterning onset in the extending vertebrate body axis.. Bioessays.

[8] Maden M, Gale E, Kostetskii I, Zile M (1996). Vitamin A-deficient quail embryos have half a hindbrain and other neural defects.. Curr Biol.

[9] Wilson L, Gale E, Chambers D, Maden M (2004). Retinoic acid and the control of dorsoventral patterning in the avian spinal cord.. Dev Biol.

[10] Jacobs S, Lie DC, DeCicco KL, Shi Y, DeLuca LM (2006). Retinoic acid is required early during adult neurogenesis in the dentate gyrus.. Proc Natl Acad Sci U S A.

[11] Novitch BG, Wichterle H, Jessell TM, Sockanathan S (2003). A requirement for retinoic acid-mediated transcriptional activation in ventral neural patterning and motor neuron specification.. Neuron.

[12] Zhelyaznik N, Schrage K, McCaffery P, Mey J (2003). Activation of retinoic acid signalling after sciatic nerve injury: up-regulation of cellular retinoid binding proteins.. Eur J Neurosci.

[13] Kim M, Habiba A, Doherty JM, Mills JC, Mercer RW (2009). Regulation of mouse embryonic stem cell neural differentiation by retinoic acid.. Dev Biol.

[14] Soprano DR, Teets BW, Soprano KJ (2007). Role of retinoic acid in the differentiation of embryonal carcinoma and embryonic stem cells.. Vitam Horm.

[15] Martins AH, Resende RR, Majumder P, Faria M, Casarini DE (2005). Neuronal differentiation of P19 embryonal carcinoma cells modulates kinin B2 receptor gene expression and function.. J Biol Chem.

[16] Hirami Y, Osakada F, Takahashi K, Okita K, Yamanaka S (2009). Generation of retinal cells from mouse and human induced pluripotent stem cells.. Neurosci Lett.

[17] Yurov YB, Iourov IY, Vorsanova SG, Liehr T, Kolotii AD (2007). Aneuploidy and confined chromosomal mosaicism in the developing human brain.. PLoS ONE.

[18] Rehen SK, McConnell MJ, Kaushal D, Kingsbury MA, Yang AH (2001). Chromosomal variation in neurons of the developing and adult mammalian nervous system.. Proc Natl Acad Sci U S A.

[19] Rajendran RS, Zupanc MM, Losche A, Westra J, Chun J (2007). Numerical chromosome variation and mitotic segregation defects in the adult brain of teleost fish.. Dev Neurobiol.

[20] Kaushal D, Contos JJ, Treuner K, Yang AH, Kingsbury MA (2003). Alteration of gene expression by chromosome loss in the postnatal mouse brain.. J Neurosci.

[21] Yurov YB, Iourov IY, Monakhov VV, Soloviev IV, Vostrikov VM (2005). The variation of aneuploidy frequency in the developing and adult human brain revealed by an interphase FISH study.. J Histochem Cytochem.

[22] Westra JW, Peterson SE, Yung YC, Mutoh T, Barral S (2008). Aneuploid mosaicism in the developing and adult cerebellar cortex.. J Comp Neurol.

[23] Kingsbury MA, Friedman B, McConnell MJ, Rehen SK, Yang AH (2005). Aneuploid neurons are functionally active and integrated into brain circuitry.. Proc Natl Acad Sci U S A.

[24] Paulsen BS, Souza CS, Chicaybam L, Bonamino MH, Bahia M (2011). Agathisflavone Enhances Retinoic Acid-Induced Neurogenesis and Its Receptors alpha and beta in Pluripotent Stem Cells.. Stem Cells Dev.

[25] (2003). Guidelines for the Care and Use of Mammals in Neuroscience and Behaviour Research..

[26] Pear WS, Nolan GP, Scott ML, Baltimore D (1993). Production of high-titer helper-free retroviruses by transient transfection.. Proc Natl Acad Sci U S A.

[27] Rossant J, McBurney MW (1982). The developmental potential of a euploid male teratocarcinoma cell line after blastocyst injection.. J Embryol Exp Morphol.

[28] Hooper M, Hardy K, Handyside A, Hunter S, Monk M (1987). HPRT-deficient (Lesch-Nyhan) mouse embryos derived from germline colonization by cultured cells.. Nature.

[29] Nichols J, Evans EP, Smith AG (1990). Establishment of germ-line-competent embryonic stem (ES) cells using differentiation inhibiting activity.. Development.

[30] Chan EM, Yates F, Boyer LF, Schlaeger TM, Daley GQ (2008). Enhanced plating efficiency of trypsin-adapted human embryonic stem cells is reversible and independent of trisomy 12/17.. Cloning Stem Cells.

[31] Rebuzzini P, Neri T, Zuccotti M, Redi CA, Garagna S (2008). Chromosome number variation in three mouse embryonic stem cell lines during culture.. Cytotechnology.

[32] Mayshar Y, Ben-David U, Lavon N, Biancotti JC, Yakir B (2010). Identification and classification of chromosomal aberrations in human induced pluripotent stem cells.. Cell Stem Cell.

[33] Resende RR, Gomes KN, Adhikari A, Britto LR, Ulrich H (2008). Mechanism of acetylcholine-induced calcium signaling during neuronal differentiation of P19 embryonal carcinoma cells in vitro.. Cell Calcium.

[34] Resende RR, Britto LR, Ulrich H (2008). Pharmacological properties of purinergic receptors and their effects on proliferation and induction of neuronal differentiation of P19 embryonal carcinoma cells.. Int J Dev Neurosci.

[35] Bibel M, Richter J, Lacroix E, Barde YA (2007). Generation of a defined and uniform population of CNS progenitors and neurons from mouse embryonic stem cells.. Nat Protoc.

[36] Bibel M, Richter J, Schrenk K, Tucker KL, Staiger V (2004). Differentiation of mouse embryonic stem cells into a defined neuronal lineage.. Nat Neurosci.

[37] Campos PB, Sartore RC, Abdalla SN, Rehen SK (2009). Chromosomal spread preparation of human embryonic stem cells for karyotyping.. J Vis Exp.

[38] Capparelli R, Cottone C, D'Apice L, Viscardi M, Colantonio L (1997). DNA content differences in laboratory mouse strains determined by flow cytometry.. Cytometry.

[39] Gupta RK, Srinivas UK (2008). Heat shock induces chromosomal instability in near-tetraploid embryonal carcinoma cells.. Cancer Biol Ther.

[40] Norppa H, Falck GC (2003). What do human micronuclei contain?. Mutagenesis.

[41] Pratt MA, Niu MY, Renart LI (2006). Regulation of survivin by retinoic acid and its role in paclitaxel-mediated cytotoxicity in MCF-7 breast cancer cells.. Apoptosis.

[42] Burgess A, Wigan M, Giles N, Depinto W, Gillespie P (2006). Inhibition of S/G2 phase CDK4 reduces mitotic fidelity.. J Biol Chem.

[43] Yang AH, Kaushal D, Rehen SK, Kriedt K, Kingsbury MA (2003). Chromosome segregation defects contribute to aneuploidy in normal neural progenitor cells.. J Neurosci.

[44] Sareen D, McMillan E, Ebert AD, Shelley BC, Johnson JA (2009). Chromosome 7 and 19 trisomy in cultured human neural progenitor cells.. PLoS One.

[45] Hong Y, Stambrook PJ (2004). Restoration of an absent G1 arrest and protection from apoptosis in embryonic stem cells after ionizing radiation.. Proc Natl Acad Sci U S A.

[46] Aladjem MI, Spike BT, Rodewald LW, Hope TJ, Klemm M (1998). ES cells do not activate p53-dependent stress responses and undergo p53-independent apoptosis in response to DNA damage.. Curr Biol.

[47] Stead E, White J, Faast R, Conn S, Goldstone S (2002). Pluripotent cell division cycles are driven by ectopic Cdk2, cyclin A/E and E2F activities.. Oncogene.

[48] Fluckiger AC, Marcy G, Marchand M, Negre D, Cosset FL (2006). Cell cycle features of primate embryonic stem cells.. Stem Cells.

[49] Herget T, Specht H, Esdar C, Oehrlein SA, Maelicke A (1998). Retinoic acid induces apoptosis-associated neural differentiation of a murine teratocarcinoma cell line.. J Neurochem.

[50] Andollo N, Boyano MD, Andrade R, Zalduendo MM, Eguizabal C (2005). Structural and functional preservation of specific sequences of DNA and mRNA in apoptotic bodies from ES cells.. Apoptosis.

[51] Okazawa H, Shimizu J, Kamei M, Imafuku I, Hamada H (1996). Bcl-2 inhibits retinoic acid-induced apoptosis during the neural differentiation of embryonal stem cells.. J Cell Biol.

[52] Decordier I, Cundari E, Kirsch-Volders M (2008). Survival of aneuploid, micronucleated and/or polyploid cells: crosstalk between ploidy control and apoptosis.. Mutat Res.

[53] Damelin M, Sun YE, Sodja VB, Bestor TH (2005). Decatenation checkpoint deficiency in stem and progenitor cells.. Cancer Cell.

[54] Makrantoni V, Stark MJ (2009). Efficient chromosome biorientation and the tension checkpoint in Saccharomyces cerevisiae both require Bir1.. Mol Cell Biol.

[55] Sun SC, Wei L, Li M, Lin SL, Xu BZ (2009). Perturbation of survivin expression affects chromosome alignment and spindle checkpoint in mouse oocyte meiotic maturation.. Cell Cycle.

[56] Lens SM, Wolthuis RM, Klompmaker R, Kauw J, Agami R (2003). Survivin is required for a sustained spindle checkpoint arrest in response to lack of tension.. Embo J.

[57] Li F, Ackermann EJ, Bennett CF, Rothermel AL, Plescia J (1999). Pleiotropic cell-division defects and apoptosis induced by interference with survivin function.. Nat Cell Biol.

[58] Saito T, Hama S, Izumi H, Yamasaki F, Kajiwara Y (2008). Centrosome amplification induced by survivin suppression enhances both chromosome instability and radiosensitivity in glioma cells.. Br J Cancer.

